# Long-term metabolic consequences of acute dioxin exposure differ between male and female mice

**DOI:** 10.1038/s41598-020-57973-0

**Published:** 2020-01-29

**Authors:** Myriam P. Hoyeck, Hannah Blair, Muna Ibrahim, Shivani Solanki, Mariam Elsawy, Arina Prakash, Kayleigh R. C. Rick, Geronimo Matteo, Shannon O’Dwyer, Jennifer E. Bruin

**Affiliations:** 1 Department of Biology & Institute of Biochemistry, Carleton University, Ottawa, ON Canada; 2 Laboratory of Molecular and Cellular Medicine, Department of Cellular & Physiological Sciences, University of British Columbia, Vancouver, Canada

**Keywords:** Cell biology, Mechanisms of disease, Endocrine system and metabolic diseases, Metabolic disorders

## Abstract

Epidemiological studies have consistently shown an association between exposure to environmental pollutants and diabetes risk in humans. We have previously shown that direct exposure of mouse and human islets (endocrine pancreas) to the highly persistent pollutant TCDD (2,3,7,8-tetrachlorodibenzo-*p*-dioxin) causes reduced insulin secretion *ex vivo*. Furthermore, a single high-dose of TCDD (200 µg/kg) suppressed both fasting and glucose-induced plasma insulin levels and promoted beta-cell apoptosis after 7 days in male mice. The current study investigated the longer-term effects of a single high-dose TCDD injection (20 µg/kg) on glucose metabolism and beta cell function in male and female C57Bl/6 mice. TCDD-exposed males displayed modest fasting hypoglycemia for ~4 weeks post-injection, reduced fasting insulin levels for up to 6 weeks, increased insulin sensitivity, decreased beta cell area, and increased delta cell area. TCDD-exposed females also had long-term suppressed basal plasma insulin levels, and abnormal insulin secretion for up to 6 weeks. Unlike males, TCDD did not impact insulin sensitivity or islet composition in females, but did cause transient glucose intolerance 4 weeks post-exposure. Our results show that a single exposure to dioxin can suppress basal insulin levels long-term in both sexes, but effects on glucose homeostasis are sex-dependent.

## Introduction

Diabetes mellitus is one of the leading causes of death worldwide, and its prevalence is increasing at alarming rates^1^. Type 2 diabetes (T2D) is the most common form of diabetes, and is characterized by chronic hyperglycemia, peripheral insulin resistance, and insufficient insulin production by pancreatic beta cells^2^. The rapid increase in diabetes incidence worldwide cannot be accounted for by genetics and lifestyle alone, suggesting that other environmental factors are likely contributing to diabetes etiology. In particular, epidemiological studies have reported an association between exposure to persistent organic pollutants (POPs) and increased diabetes incidence^3,4^. However, while epidemiological studies can uncover correlative associations, basic research in model systems is required to investigate causation.

Generally, POPs are lipophilic chemicals that resist degradation, leading to widespread global dispersion, bioaccumulation, and long-term release into the environment^5^. Dioxins and dioxin-like compounds are a broad class of POPs that activate the aryl hydrocarbon receptor (AhR), which induces a variety of target genes, including cytochrome P450 *(Cyp)1a1* and *Cyp1a2*. Although CYP enzymes are essential for xenobiotic metabolism, CYP-mediated substrate oxidation can also generate highly reactive intermediate metabolites that cause oxidative stress and DNA/protein damage^6^. Our lab has shown that CYP1A enzymes are inducible and functional in human and mouse pancreatic islets, and that these enzymes remain active for at least two weeks following a single acute exposure to the classic dioxin, 2,3,7,8-tetrachlorodibenzo-*p*-dioxin (TCDD)^7^. These data indicate that dioxins reach the endocrine pancreas *in vivo*, which may have important implications for islet cell function and survival.

Epidemiological studies have linked serum concentrations of dioxin/dioxin-like chemicals with both increased diabetes incidence^8–15^ and decreased insulin secretion in humans^16,17^. Suppressed insulin secretion was also observed in rodent beta cell lines after direct exposure to organochlorine pesticides and dioxin-like polychlorinated biphenyls (PCBs) *in vitro*^16,18^, and in isolated rat and mouse islets 24-hours following *in vivo* exposure to TCDD^19^. Our lab has also shown that direct exposure of mouse and human islets to TCDD for 48-hours *ex vivo* causes reduced insulin secretion^7^. Furthermore, a single high-dose injection of TCDD (200 µg/kg) in male mice led to reduced plasma insulin levels for 2 weeks *in vivo*, and impaired glucose-stimulated insulin secretion by isolated islets *ex vivo* after 1 week^7^. We also observed a substantial increase in beta cell apoptosis in these mice, suggesting that TCDD affects both beta cell function and survival^7^. However, this high dose of TCDD caused significant weight loss and severe hypoglycemia after 2 weeks, which prevented us from assessing longer-term metabolic effects of transient TCDD exposure *in vivo*.

Another limitation of our previous study was that we only examined male mice, similar to other groups^18,20–22^. To our knowledge, there are no published studies investigating whether dioxin exposure leads to sex-specific metabolic outcomes in rodents. Interestingly, epidemiological studies suggest a possible sex difference in the association between diabetes incidence and serum POP levels. Although studies are contradictory, there is evidence suggesting a stronger association between dioxin exposure and diabetes incidence in women compared to men^9,11,23^. However, there are limited epidemiological studies that investigate sex differences in this context and they often lack sufficient power to be conclusive, emphasising the need to further study sex-dependent metabolic effects of dioxin exposure in a carefully controlled setting. The purpose of this study was to investigate the long-term implications of a single exposure to TCDD on glucose homeostasis and islet cell physiology in male and female mice, using a lower dose than in our previous work.

## Results

### A single dose of TCDD causes a long-term reduction of plasma insulin levels in male mice

**Figure 1:**
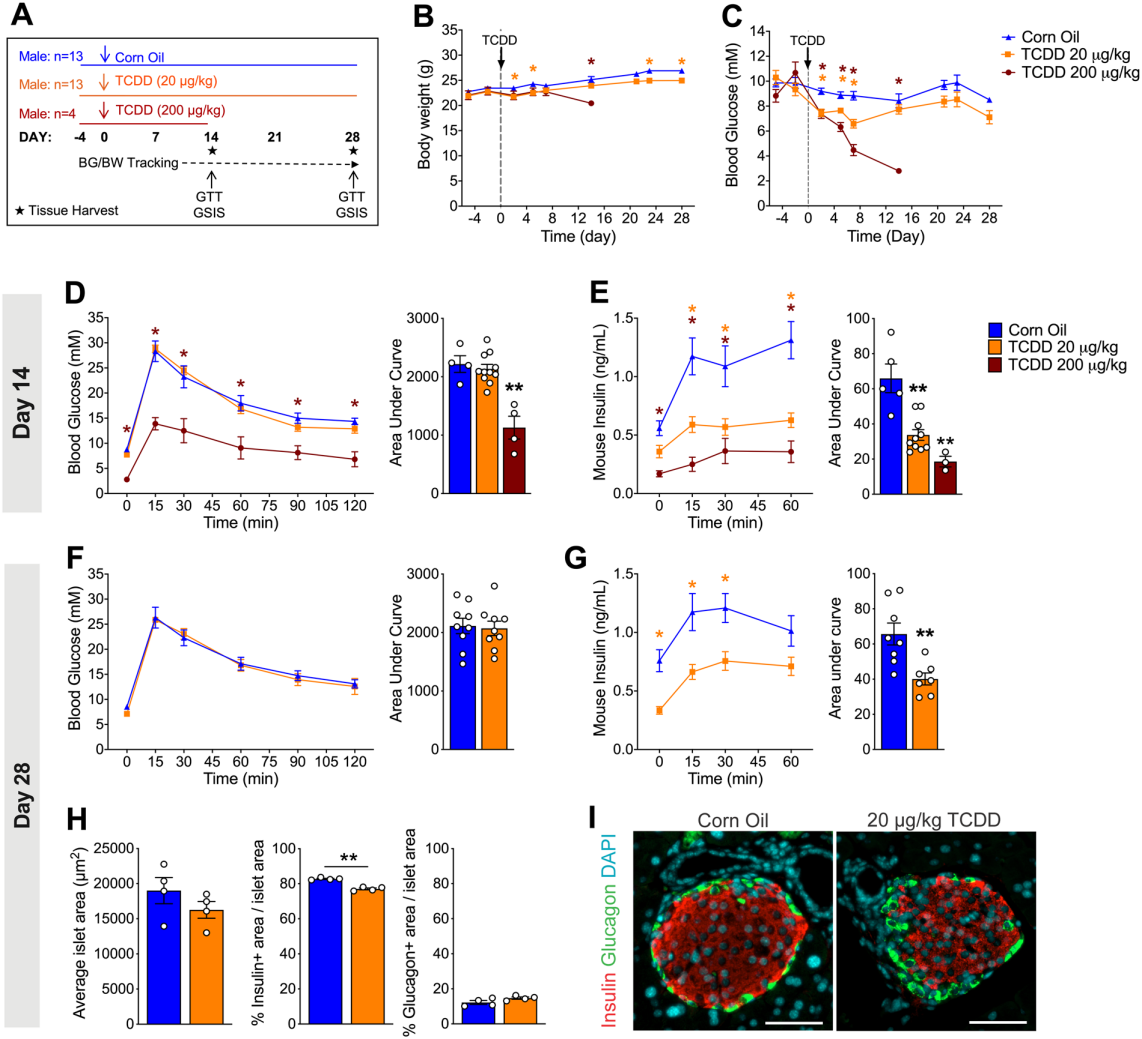
Long-term reduction in plasma insulin levels, but normal glucose tolerance *in vivo* after a single 20 µg/kg dose of TCDD in male mice. (**A**) Schematic summary timeline of the study. Male mice were injected with either corn oil, 20 μg/kg TCDD, or 200 μg/kg TCDD on day 0 and followed for up to 4 weeks. BG = blood glucose; BW = body weight; GTT = glucose tolerance test; GSIS = glucose-stimulated insulin secretion. **(B)** Body weight and **(C)** blood glucose levels were measured after a morning fast throughout the study. **(D,F)** Blood glucose and **(E,G)** plasma insulin levels were measured during an i.p. GTT on day 14 **(D,E)** and day 28 **(F,G)**. **(H)** Average islet area, and % of islet area that is immunoreactive for either insulin or glucagon. **(I)** Representative images of paraffin-embedded pancreas sections on day 28 after exposure to control or TCDD (20 μg/kg). Tissues are stained for insulin (red), glucagon (green), and DAPI (blue). Scale bars = 50 μm. All data are presented as mean ± SEM. Individual data points on bar graphs represent biological replicates (different mice). *p<0.05, **p<0.01 versus control. The following statistical tests were used: (**B,C**) days −5 to 14, two-way ANOVA with Dunnett test; day 21 to 28, two-way ANOVA with Sidak test; (**D,E**) line graphs: two-way RM ANOVA with Dunnett test; bar graphs, one-way ANOVA with Dunnett test; (**F,G,H**) line graphs, two-way RM-ANOVA with Sidak test; bar graphs, two-tailed unpaired t-test.

To examine longer-term effects of acute TCDD exposure on glucose homeostasis we first tested whether the previously observed decrease in plasma insulin levels following a high dose TCDD injection (200 µg/kg)^7^ would be maintained with a lower dose of TCDD (20 µg/kg) in male mice. Furthermore, it was critical to find a dose that did not cause TCDD-induced lethality and wasting syndrome (i.e. severe weight loss and dramatic hypoglycemia)^24^ so the mice could be maintained long-term (see Fig. 1A for study timeline). Consistent with our previous study^7^, 200 µg/kg TCDD induced a significant decline in body weight and a dramatic decrease in blood glucose levels to ~3 mM within 12 days following exposure (Fig. 1B,C). In contrast, 20 µg/kg TCDD only modestly decreased body weight, and fasting blood glucose remained within a normal physiological range throughout the study (>6 mM; Fig. 1B,C). During a glucose tolerance test (GTT) at 2 weeks post-injection, mice exposed to 200 µg/kg TCDD showed severe hypoglycemia (Fig. 1D) and a dramatic decrease in plasma insulin levels (Fig. 1E). Interestingly, 20 µg/kg TCDD had no effect on glucose tolerance at either 2 or 4 weeks post-exposure (Fig. 1D,F), but significantly reduced plasma insulin levels during the glucose challenge at both time points (Fig. 1E,G). The effect of TCDD on insulin secretion was clearly dose-dependent at 2 weeks post-exposure (Fig. 1E). The long-term suppression of plasma insulin levels in mice exposed to 20 µg/kg TCDD was associated with a significant decrease in insulin^+^ area per islet at 4 weeks (Fig. 1H,I). There was no significant change in glucagon^+^ area or overall islet size (Fig. 1H,I).

We further validated the 20 µg/kg TCDD dosing model by ensuring that TCDD was reaching the endocrine pancreas, using *Cyp1a1* induction as a biomarker for direct *in vivo* exposure^7^. At 2–4 weeks following the 20 µg/kg or 200 µg/kg TCDD injection, *Cyp1a1* expression was induced ~33-fold and ~25-fold, respectively, in the liver (Fig. S1A) compared to ~3-fold and ~12-fold, respectively, in the islets (Fig. S1B). Moreover, we also observed a dose-dependent effect of TCDD on CYP1A1 enzyme activity in islets (Fig. S1C), indicating that TCDD reaches the islets and induces long-term activation of CYP1A1 with both doses. We also verified that 20 µg/kg TCDD was not overtly cytotoxic by measuring inflammatory and apoptotic pathways in the liver and islets at 2 weeks post-TCDD. Consistent with our previous findings^7^, we observed an increase in pro-inflammatory cytokines, *Tnfa and Il-1b*, and the anti-apoptotic gene *Birc3* in the liver (Fig. S1D), along with a decrease in *Il-1b* and *Birc3* in the islets (Fig. S1E) at 2 weeks following 200 µg/kg TCDD. The 20 µg/kg TCDD dose caused similar and dose-dependent changes in liver inflammation pathways (Fig. S1D), but no changes in islets (Fig. S1E).

### TCDD suppresses fasting insulin levels long-term in both males and females, but effects on glycemia, body weight, and insulin tolerance are sex-dependent

**Figure 2:**
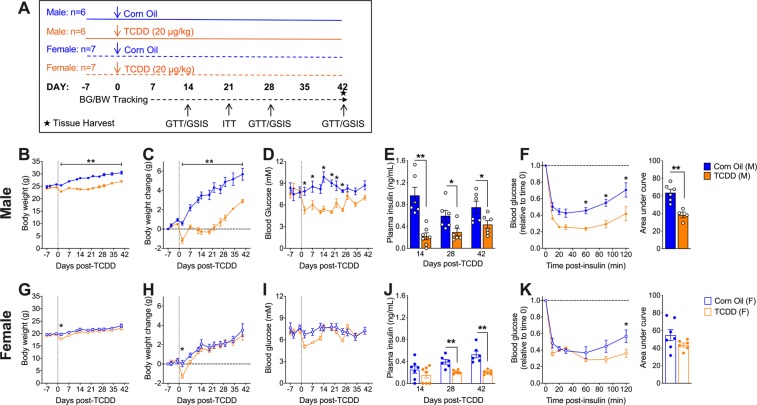
Sex differences in fasting metabolic phenotype and insulin sensitivity following TCDD exposure. (**A)** Schematic summary timeline of the study. (**B–F**) Male and (**G–K**) female mice were injected with either corn oil or 20 μg/kg TCDD on day 0 and followed for up to 6 weeks. BG = blood glucose; BW = body weight; ITT = insulin tolerance test; GTT = glucose tolerance test; GSIS = glucose-stimulated insulin secretion. **(B,C,G,H)** Body weight was measured throughout the study and data are expressed as raw values **(B,G)**, and change in body weight relative to the first measurement on day -7 **(C,H)**. **(D,I)** Blood glucose and **(E,J)** plasma insulin levels were measured after a 4 hour morning fast throughout the study. **(F,K)** Blood glucose levels during an ITT on day 21 (values are normalized relative to time 0 for each mouse). All data are presented as mean ± SEM. Individual data points on bar graphs represent biological replicates (different mice). *p<0.05, **p<0.01 versus control. The following statistical tests were used: **(B–D,G–I)** two-way ANOVA with Sidak test; **(E,F)** bar graphs, unpaired two**-**tailed t-test; **(J,K)** bar graphs, non-parametric Mann-Whitney test; **(F,K)** line graphs, two-way RM-ANOVA with Sidak test.

We next investigated whether the long-term effects of TCDD (using only the 20 µg/kg dose from this point forward) on basal metabolism and fasting insulin levels are sex dependent (see Fig. 2A for study timeline). Consistent with the first cohort (Fig. 1B,C), TCDD-exposed male mice showed a sustained decrease in body weight throughout the 6-week study (Fig. 2B,C) and reduced fasting blood glucose levels from days 2 to 26 post-injection (Fig. 2D). Male mice also had significantly reduced fasting plasma insulin levels at 2, 4, and 6 weeks post-TCDD (Fig. 2E), and a pronounced increase in insulin sensitivity at 3 weeks (Fig. 2F). In contrast, female mice only had a transient and very modest decrease in body weight (Fig. 2G,H) and fasting blood glucose levels (Fig. 2I) in the first week post-TCDD. Interestingly, TCDD-exposed females had significantly reduced plasma insulin levels at 4 and 6 weeks (Fig. 2J), but no overall change in insulin sensitivity at 3 weeks (Fig. 2K).

### TCDD induces sex-dependent changes in glucose tolerance and insulin secretion

**Figure 3:**
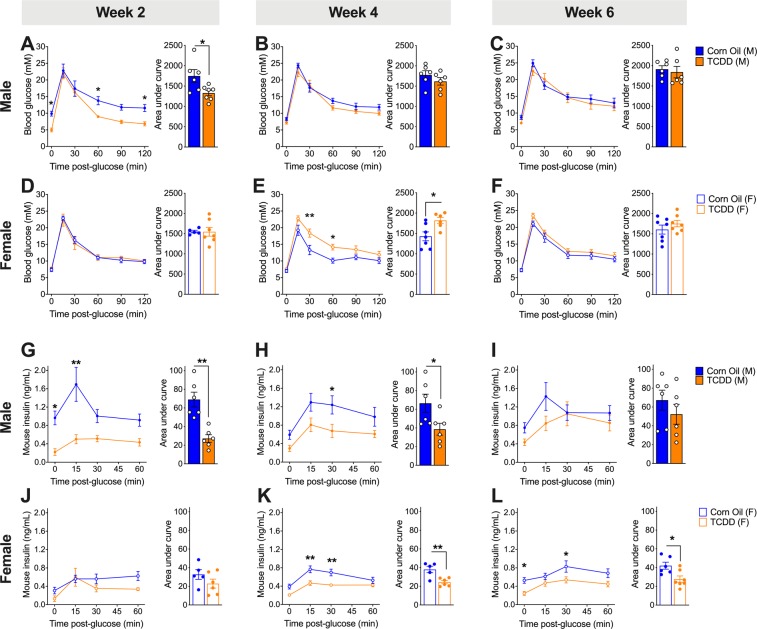
Transient TCDD exposure leads to long-term suppression of plasma insulin in both sexes, but sex differences in glucose tolerance *in vivo*. (**A–C, G–I)** Male and **(D–F,J–L)** female mice were injected with either corn oil or 20 μg/kg TCDD on day 0 and glucose tolerance and glucose-stimulated insulin secretion were assessed *in vivo* on days 14, 28, and 42 (see Fig. 2A for schematic timeline). **(A–F)** Blood glucose levels during a GTT on days 14 **(A,D)**, 28 **(B,E)**, and 42 **(C,F)**. **(G–L)** Plasma insulin levels during the GTT on days 14 **(G,J)**, 28 **(H,K)**, and 42 **(I,L)**. All data are presented as mean ± SEM. Individual data points on bar graphs represent biological replicates (different mice). *p < 0.05, **p < 0.01 versus control. The following statistical tests were used: (**A–L**) line graphs, two-way RM-ANOVA with Sidak test; bar graphs, unpaired two-tailed t-test, except for (**D**) which was analyzed by a non-parametric Mann-Whitney test. See Fig. S2 for additional data from a second cohort.

To further characterize sex-dependent effects of TCDD exposure on metabolism, we assessed glucose tolerance and beta cell function for 6 weeks *in vivo* after a single TCDD injection. Male mice were modestly hypoglycemic during a glucose challenge at 2 weeks post-TCDD compared to controls, but their glycemia was restored by 4 weeks (Fig. 3A,B,C). Male mice also showed a significant decrease in plasma insulin levels during GTTs at 2 and 4 weeks post-TCDD, with levels returning to normal by 6 weeks (Fig. 3G,H,I). Similar trends were seen in a separate cohort of male mice, where plasma insulin levels were suppressed at 1 and 2 weeks post-TCDD but normal by 4 weeks (Fig. S2A,B,C). Glucose tolerance was also normal in TCDD-exposed males at 4 weeks (Fig. S2D).

In contrast, female mice had normal glucose tolerance at 2 and 6 weeks post-TCDD (Fig. 3D,F**)**, but were significantly hyperglycemic during a GTT at 4 weeks post-TCDD (Fig. 3E). Importantly, this transient hyperglycemia at 4 weeks post-TCDD was replicated in a second cohort of female mice (Fig. S2H**)**. TCDD-exposed females showed normal plasma insulin levels during a glucose challenge at 2 weeks post-injection, but had significantly decreased insulin levels at 4 and 6 weeks compared to controls (Fig. 3J,K,L). In the second cohort, females were transiently hyperinsulinemic at 1 week post-TCDD, followed by reduced insulin levels at 2 weeks, and normal levels by 4 weeks (Fig. S2E,F,G).

### Islet composition is altered in male but not female mice following TCDD exposure

**Figure 4:**
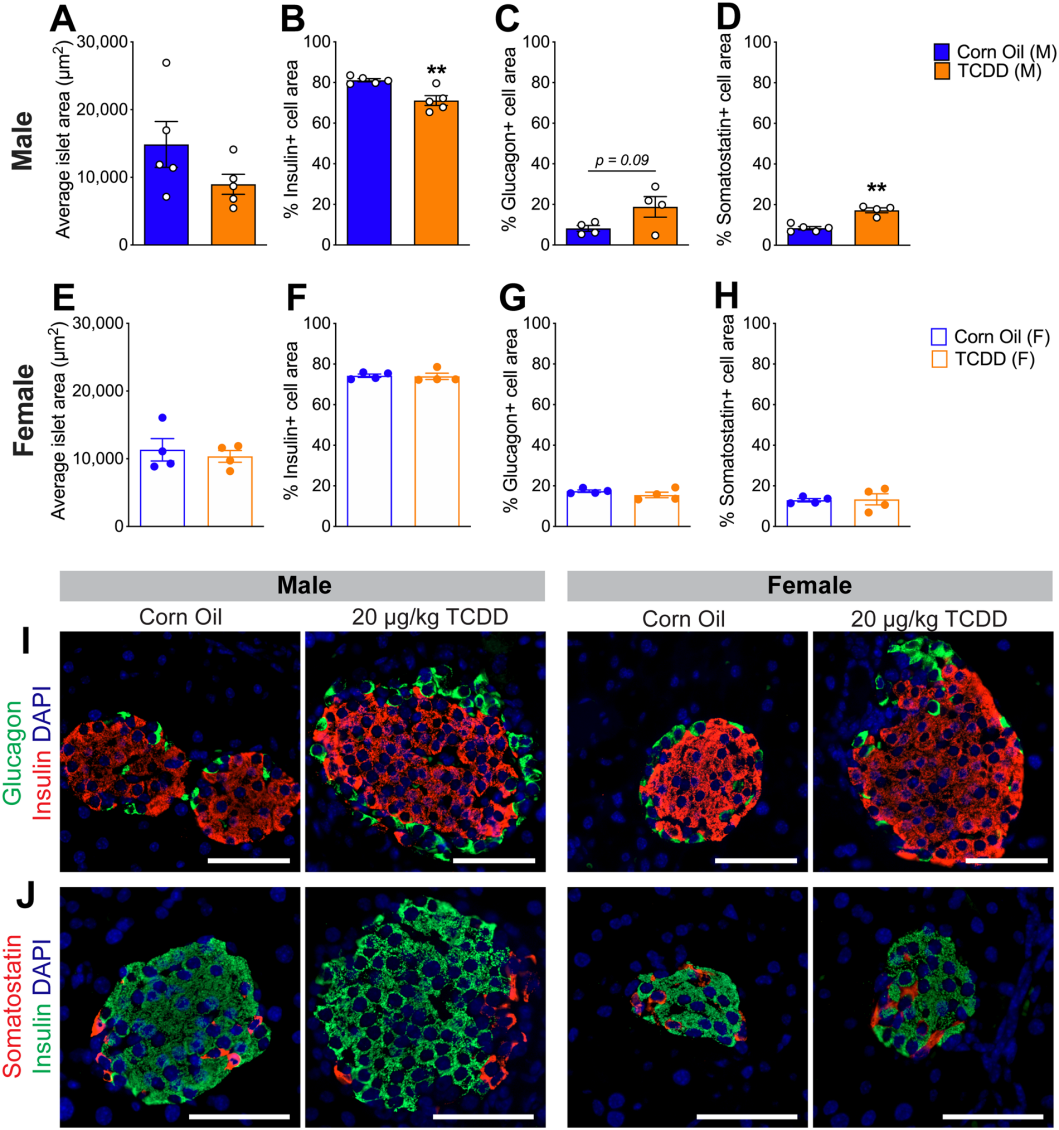
TCDD exposure decreases % beta cell area and increases % delta cell area in male mice but does not affect islet cell composition in female mice. (**A–D)** Male and **(E–H)** female mice were injected with either corn oil or 20 μg/kg TCDD on day 0 and pancreas tissue was harvested on day 42 for analysis by immunofluorescence staining (see Fig. 2A for schematic timeline). **(A,E)** Average islet area. **(B–D,F-H)** % of islet area that is immunoreactive for insulin (**B,F)**, glucagon **(C,G)**, or somatostatin **(D,H)**. **(I–J)** Representative images of pancreas sections from male and female mice exposed to either corn oil or TCDD showing immunofluorescence staining for insulin/glucagon or insulin/somatostatin. Scale bars = 50 μm. All data are presented as mean ± SEM. Individual data points on bar graphs represent biological replicates (different mice). **p<0.01 versus control; all data was analyzed by unpaired two-tailed t-test.

Finally, we assessed islet composition to determine whether the decreased beta cell area observed in TCDD-exposed male mice (Fig. 1H,I) was reproducible and whether there was sexual dimorphism in beta cell survival. Consistent with the first cohort of male mice (Fig. 1H,I**)**, we again observed a modest but significant decrease (~10%) in the % insulin^+^ area per islet in TCDD-exposed males (Fig. 4B,I,J**)**, a trend towards increased % glucagon^+^ area (Fig. 4C,I), a significant increase in % somatostatin^+^ area (Fig. 4D,J), and no overall change in average islet size (Fig. 4A**)**. Interestingly, no changes in endocrine cell composition were observed in the females at 6 weeks after TCDD exposure (Fig. 4E–J).

## Discussion

This study provides clear evidence that TCDD exposure causes sex-specific metabolic disruption in mice. A single exposure to this persistent pollutant suppressed basal insulin levels in both male and female mice for up to 6 weeks *in vivo*, although this was consistently delayed in females compared to males. Additionally, the overall impact on metabolism differed drastically between sexes, whereby TCDD-exposed males showed loss of beta cell mass, increased insulin sensitivity, weight loss, and hypoglycemia, while females became transiently hyperglycemic but had either minimal or no changes to islet composition, insulin sensitivity and body weight. These data highlight the need to investigate the link between pollutant exposure and diabetes risk in both sexes, as the mechanisms of action are most likely sex-specific.

A significant association between dioxin exposure and diabetes incidence has been reported in numerous epidemiological studies^8–13,25–27^, but this is not always consistent^28–30^. For example, diabetes incidence was increased in Vietnam veterans^12^ and the Seveso cohort^11^ exposed to TCDD and in the Yucheng cohort exposed to dioxin-like PCBs^9^, but there was no significant association between diabetes incidence and serum TCDD levels in the NIOSH cohort^28^. Interestingly, recent studies suggest that other factors, such as obesity and diet, may contribute to the association between diabetes and POP exposure, yet are often not considered in epidemiological studies^31,32^. Additionally, most studies have not reported sex-specific associations between POP exposure and diabetes, either because they only included one sex^12,25,33,34^ or their results were not stratified according to sex^14,35–38^. The few studies that delineated effects by sex have suggested that the link between diabetes incidence and POP exposure may be stronger in females than males^9,11^. For example, in both the TCDD-exposed Seveso population and the PCB-exposed Yucheng cohort, the increased risk of diabetes was only seen in females^9,11^. Studies on non-persistent pollutants have also reported sex-dependent effects on diabetes incidence. Air pollution (including NO_2_, particulate matter, and SO_2_) was associated with an increased risk of diabetes in women but not in men^39,40^, whereas NO_X_ and O_3_ was associated with diabetes in both men and women, but the association was stronger in women^41^. Based on these studies, we predicted that environmental pollutants would likely disrupt metabolism in a sex-dependent manner, and that females may be more susceptible to metabolism-disrupting pollutants than males. However, mechanistic studies in a well-controlled model system are clearly required to elucidate potential causal effects of pollutants and to better understand sex-specific mechanisms of action.

To investigate causality, we assessed the long-term metabolic impact of transient TCDD exposure in a mouse model. We had previously observed that exposure to a single high dose of TCDD (200 µg/kg) reduced plasma insulin levels for 2 weeks and caused hypoglycemia in male mice^7^, effects that were reproduced in the current study. These data indicate that TCDD disrupts glucose homeostasis and beta cell function, although the hypoglycemia in male mice did not necessarily support our hypothesis of a causal association between TCDD and increased diabetes risk. However, TCDD-exposed male mice also had a dramatic 22-fold increase in beta cell apoptosis after 1 week^7^, suggesting that TCDD may have irreversible effects on beta cell mass. In this study we opted to use a lower dose of TCDD to allow for longer term metabolic tracking and pancreas analysis. Interestingly, 20 µg/kg TCDD suppressed plasma insulin levels in both males and females for up to 6 weeks, beyond when meaningful levels of TCDD would be expected to remain in the pancreas. For example, only ~0.03% of a 10 µg/kg TCDD dose remained in the pancreas after 5 weeks in female B6C3F1 mice^42^. Therefore, we speculate that TCDD may have long-lasting effects on beta cell function that persist after the chemical has been cleared from pancreas tissue.

Although TCDD-exposed male mice did not develop hyperglycemia in this study (in fact, the opposite occurred), the reduction in beta cell mass could be a concern longer term as endogenous beta cell regeneration is extremely limited, especially in humans. However, in the 6-week timeframe of this study, male mice compensated for insulin insufficiency by increasing insulin sensitivity to maintain glucose homeostasis. In contrast, females did not adequately adjust their insulin sensitivity, resulting in transient hyperglycemia coinciding with hypoinsulinemia, which is characteristic of diabetes progression. The females returned to normoglycemia roughly when we expect the majority of TCDD to have been excreted. Taken together, we predict that an environment with ongoing pollutant exposure might be particularly problematic for females, given their impaired metabolic adaptation to insulin insufficiency. Our data also suggest that TCDD exposure alone may be insufficient to cause diabetes but could influence diabetes susceptibility in both males and females, at least in part by altering insulin secretion and/or causing irreversible beta cell death.

The mechanisms underlying TCDD-induced sex differences remain to be investigated but there are several possibilities to consider. We noticed that the onset of reduced plasma insulin levels following TCDD exposure was consistently delayed in females compared to males. These data suggest that TCDD may be absorbed, distributed, or metabolised differently in females and males. Unfortunately, there is currently a gap in the literature assessing sex dimorphisms in the kinetics of TCDD metabolism, particularly in the pancreas, preventing us from further elaborating on the subject. It is also possible that TCDD may interfere with sex hormones, specifically estrogen, leading to sex-specific changes in metabolism. Indeed, diabetes prevalence is generally higher in men than women, which has been attributed to a protective role of estrogen on body composition and glucose homeostasis^43^. Previous studies have reported crosstalk between AhR and estrogen signaling^44^, although the role of AhR in metabolism is complex and not fully understood^45^. Whether the TCDD-AhR complex interferes with the protective effects of estrogen in our study remains to be investigated but could explain why only female mice develop hyperglycemia following TCDD exposure.

It is important to note limitations in our study that may confound the interpretation of our results. First, extensive studies have shown that sex-specific sensitivity to dioxins display wide inter- and intraspecies differences^46–49^. As such, more studies are required to determine if the sex-specific effects of TCDD on metabolism are similar in other strains and species besides C57BL/6 mice. Second, the dose used in this study is not physiologically relevant to the general population. Human daily intake is approximately 0.3–3.0 pg/kg of body weight^50^, which would be achieved with chronic dosing of ~20 ng/kg TCDD rather than an acute exposure of 20 µg/kg as was used in this study. As such this study serves more as a proof-of-principle that TCDD has the potential to affect glucose homeostasis and beta cell biology in a sex-dependent manner. We are currently investigating whether similar effects are observed following chronic low-dose exposure to TCDD.

Dioxin and dioxin-like pollutants have consistently been associated with an increase in diabetes risk in humans^3,4^. However, research thus far has mainly investigated the acute effects of TCDD on metabolism in male rodents. We have shown here that a single transient exposure to TCDD has long-lasting effects on beta cell function and glucose tolerance *in vivo*. Furthermore, TCDD has sex-dependent effects on metabolism, whereby only male mice experience beta cell loss and only female mice become hyperglycemic following TCDD exposure. These results emphasize the need to study the effect of environmental pollutants on metabolism in both sexes and that studies in males should not be used to predict the effects of pollutants in females. It will be particularly interesting to investigate whether the loss of beta cell mass in males or the transient hyperglycemia observed in females after a single high dose of TCDD might progress into overt diabetes with an additional metabolic challenge. Further research is also required to elucidate the underlying mechanism of dioxin-induced changes in metabolism and to determine if these changes occur with a physiologically relevant chronic dosing model.

## Methods

### Animals

All mice received *ad libitum* access to a standard irradiated diet (Harlan Laboratories, Teklad Diet #2918, Madison, WI) and were maintained on a 12 hour light/dark cycle throughout the study. All experiments were approved by the University of British Columbia or Carleton University Animal Care Committees and carried out in accordance with the Canadian Council on Animal Care guidelines.

Cohort 1 **(**Figs. 1, S1): As outlined in Fig. 1A, 8 week old male C57Bl/6 mice (Jackson Labs) received a single i.p. injection of corn oil (25 ml/kg, vehicle control; n = 13), 20 μg/kg TCDD (n = 13), or 200 μg/kg TCDD (n = 4). Islets were isolated from a subset of mice on days 14 (n = 4 per group) and 28 (n = 4 per group; corn oil and 20 μg/kg TCDD mice only) for RNA isolation and Cyp1a1 enzyme activity assays. Islets for RNA were stored in RLT buffer (Qiagen) at −80 °C. Whole pancreas and liver were harvested from a different subset of mice on day 28 (n = 5 per group; corn oil and 20 μg/kg TCDD mice only) and stored in 4% paraformaldehyde (PFA) for 24 hours, followed by long-term storage in 70% ethanol. A piece of liver was also flash frozen in liquid nitrogen and stored at −80 °C for RNA isolation.

Cohort 2 **(**Figs. 2–4)**:** As outlined in Fig. 2A, 8 week old male (n = 12) and female (n = 14) C57Bl/6 mice (bred in-house at the Modified Barrier Facility, University of British Columbia) received a single i.p. injection of corn oil (25 ml/kg, vehicle control) or 20 μg/kg TCDD. All mice were euthanized on day 42 to harvest pancreas in 4% PFA for histological analysis.

Cohort 3 (Fig. S2**)**: Male (n = 10) and female (n = 10) 8 week old C57Bl/6 mice (Charles River) received a single i.p. injection of corn oil (25 ml/kg, vehicle control) or 20 μg/kg TCDD. All mice were euthanized after 28 days.

### *In vivo* metabolic assessments

All metabolic analyses were performed in conscious, restrained mice and blood samples were collected via saphenous vein using heparinized microhematocrit tubes at the indicated time points. Blood glucose levels were measured using a handheld glucometer (Lifescan; Burnaby, Canada).

Body weight and blood glucose levels were assessed weekly or bi-weekly following a 4-hour morning fast. For all other metabolic tests, time 0 indicates the blood sample collected after fasting and prior to administration of glucose or insulin. For glucose tolerance tests (GTTs), mice received an i.p. bolus of glucose (2 g/kg; Vétoquinol) following a 6-hour morning fast. During the GTT, blood was collected at the indicated time points for measuring plasma insulin levels by ELISA (ALPCO mouse ultrasensitive insulin ELISA, #80-INSMSU-E01). For insulin tolerance tests (ITTs), mice received an i.p. bolus of insulin (0.7 IU/kg, Novolin ge Toronto; Novo Nordisk Canada #02024233) after a 4-hour morning fast.

### Mouse islet isolation & culture

Islets were isolated from mice by pancreatic duct injection with collagenase (1,000 units/ml; Sigma Aldrich #C7657) dissolved in Hanks’ balanced salt solution (HBSS: 137 mM NaCl, 5.4 mM KCl, 4.2 mM NaH_2_PO_4_, 4.1 mM KH_2_PO_4_, 10 mM HEPES, 1 mM MgCl_2_, 5 mM dextrose, pH 7.2). Pancreata were incubated at 37 °C for 12 minutes, vigorously agitated, and the collagenase reaction quenched by adding cold HBSS with 1mM CaCl_2_. The pancreas tissue was washed 3-times in HBSS + CaCl_2_ (centrifuging for 1 min at 1000 rpm in between washes) and resuspended in Ham’s F-10 (HyClone #SH30025.01 or Corning #10-070-CV) containing 0.5% bovine serum albumin (BSA; Sigma-Aldrich #10775835001), 100 units/ml penicillin, and 100 μg/ml streptomycin (Corning #30002CI). Pancreas tissue was filtered through a 70 μm cell strainer and islets were handpicked under a dissecting scope to>95% purity.

### Cytochrome P450 enzyme activity assay

Enzyme activity was measured in isolated mouse islets (50 islets per well) using the Promega P450-Glo CYP1A1 Assay (#V8752) in 96-well white-walled plates (Greiner Bio-One, # 655098) using the lytic method, as described by the manufacturer.

### Quantitative real time PCR

RNA was isolated from liver samples using the Qiagen RNeasy Mini Kit (#74104) or Trizol Reagent (Invitrogen #15596018), and mouse islets using the Qiagen RNeasy Micro Kit (#74004). DNase treatment was performed prior to cDNA synthesis with the iScript gDNA Clear cDNA Synthesis Kit (#1725035, Biorad, Mississauga, ON Canada). Quantitative real-time PCR (qPCR) was performed using the SsoFast EvaGreen Supermix (#1725200, Biorad) or SsoAdvanced Universal SYBR Green Supermix (#1725271, Biorad) and run on a CFX96 or CFX384 (Biorad). *Hprt* or *Ppia* was used as the reference gene since these genes displayed stable expression under control and treatment conditions. Data were analyzed using the ΔΔCT Method. Primer sequences are listed in Table S1.

### Immunofluorescent staining and image quantification

Paraffin sections (5 μm thickness) were prepared by the University of Ottawa Heart Institute Histology Core Facility (Ottawa, ON). Immunofluorescent staining was performed as previously described^7^. In brief, slides were deparaffinized with sequential incubation in xylene and ethanol. Heat-induced epitope retrieval was performed, and slides were incubated with Dako Serum Free Protein Block (#X090930–2; Agilent, Santa Clara, CA). Slides were incubated overnight at 4° with primary antibodies, and then incubated with secondary antibodies for 1 hr at room temperature. Coverslips were mounted with VECTASHIELD HardSet Mounting Medium with DAPI (#H-1500, Vector Laboratories) for counterstaining. The following antibodies were used: rabbit anti-insulin (C27C9, 1:200, Cell Signaling #3014), mouse anti-insulin (L6B10, 1:250, Cell Signaling # 8138BF), mouse anti-glucagon (1:1000, Sigma-Aldrich #G2654), rabbit anti-somatostatin (1:500, Sigma-Aldrich # HPA019472). For islet quantification, a minimum of 8 islets were imaged with an EVOS FL Cell Imaging System (Invitrogen). For each islet, the area was measured for the whole islet, insulin^+^ immunoreactivity, glucagon^+^ immunoreactivity, and somatostatin^+^ immunoreactivity using ImageJ software. The % hormone^+^ area was calculated as [(hormone^+^ area / islet area) × 100], and the average value was calculated for each mouse.

### Quantification and statistical analysis

All statistics were performed using GraphPad Prism software (GraphPad Software Inc., La Jolla, CA). Specific statistical tests are indicated in figures legends. Sample sizes are described in ***Animals*** and are also shown in schematic timelines (Figs. 1A, 2A). For all analyses, p < 0.05 was considered statistically significant. Statistically significant outliers were detected by a Grubbs’ test with α = 0.05. All data was tested for normality using a Shapiro-Wilk test and for equal variance using either a Brown-Forsyth test (for one-way ANOVAs) or an F test (for unpaired t tests). Parametric tests were used for all two-way ANOVAs, but normality and equal variance were tested on area under the curve values. Non-parametric statistics was used in cases where the data failed normality or equal variance tests. Data are presented as mean ± SEM. Individual data points on bar graphs are always biological replicates (i.e. different mice).

## Supplementary information

Supplementary information.

## Data Availability

The datasets generated and/or analyzed during the current study are available from the corresponding author upon reasonable request. No applicable resources were generated or analyzed during the current study.
