# Supplementary material for: Long-term metabolic consequences of acute dioxin exposure differ between male and female mice
Source: Sci Rep. 2020 Jan 29;10:1448. doi: 10.1038/s41598-020-57973-0 (PMC6989671; doi:10.1038/s41598-020-57973-0)
Supplement: Supplementary file 1 — Supplementary information. [file 41598_2020_57973_MOESM1_ESM.pdf]

<u>Table S1</u>: Primer sequences for qPCR

Target	Forward Sequence (5'-3')	Reverse Sequence (5'-3')	Amplicon Size (bp)	Amplification Efficiency (%)
PPIA	AGC TCT GAG CAC TGG AGA GA	GCC AGG ACC TGT ATG CTT TA	178	101.6
Hprt	GCT GAC CTG CTG GAT TAC AT	TTG GGG CTG TAC TGC TTA AC	242	122.4
Cyp1a1	ATC ACA GAC AGC CTC ATT GAG C	AGA TAG CAG TTG TGA CTG TGT C	139	135.0
Cyp1a2	CAA GAG GTT TAA GAC CTT CAA TGA TAA C	AAA GAT GTC ATT GAC AAT GTT GAC AAT	193	105.7
Tnfa	AGT CCG GGC AGG TCT ACT TT	ATG AAC ACC CAT TCC CTT CA	55	96.8
Nf-kb	CTC AGG AGC AGA AGT CTG GG	GCC GCT ATA TGC AGA GGT GT	145	103.2
Il-1b	GCC ACC TTT TGA CAG TGA TGA G	AGC TTC TCC ACA GCC ACA AT	186	95.4
Birc3	CCC GGA GAT CAG AGG TCA TTG	GAA AGG CGC TGT CTT GAA CC	172	138.7
Xiap	TCG GGT CAG CCT CCT TAA AC	TGG TGT CTG CAA GTA CAA AAG T	119	100.9

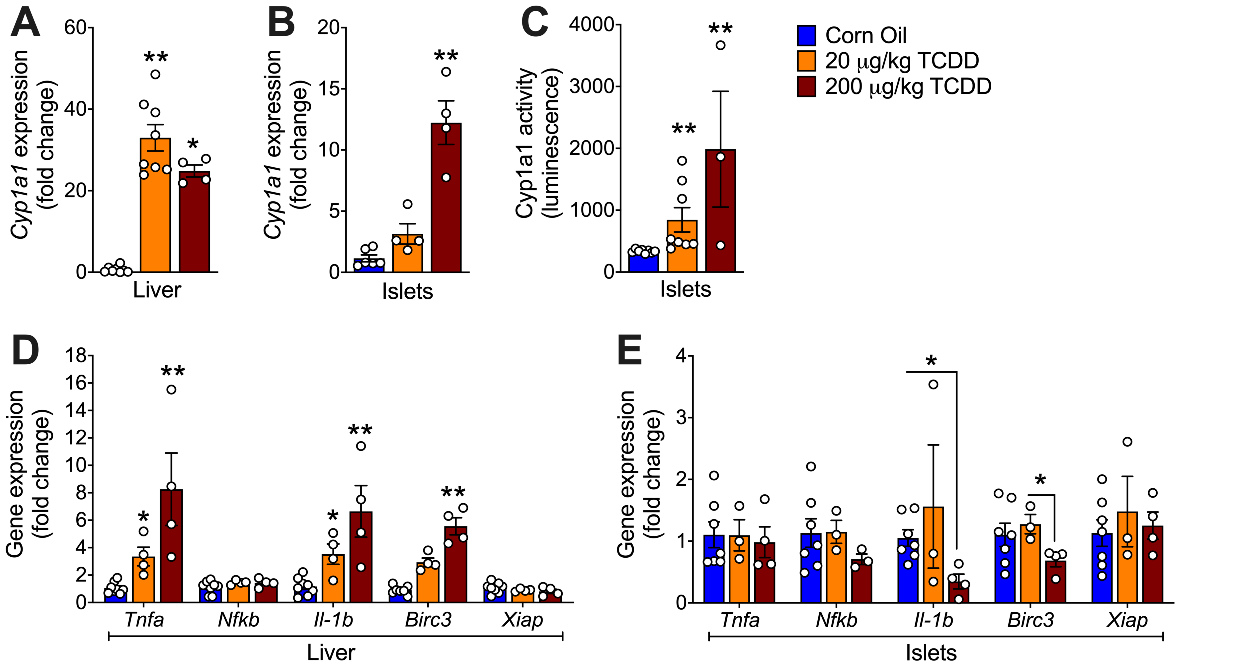

Figure S1: Islets and liver from TCDD-exposed mice have similar induction of Cyp1a1 but different stress responses. Male mice received a single injection of either corn oil, 20 μg/kg TCDD or 200 μg/kg TCDD. Tissues were collected either 2 or 4 weeks later. **(A-B)** *Cyp1a1* gene expression in liver **(A)** and islets **(B)**, expressed as fold change relative to control at 2-4 weeks. **(C)** Cyp1a1 enzyme activity in isolated islets at 2-4 weeks. **(D-E)** Expression of various genes related to inflammation and apoptosis at 2 weeks in liver **(D)** and isolated islets **(E)**. *$p<0.05$, **$p<0.01$ versus control unless indicated otherwise; all data was analyzed by a Kruskal-Wallis test with uncorrected Dunn's test for multiple comparison. All data are presented as mean ± SEM and individual data points represent biological replicates (different mice).

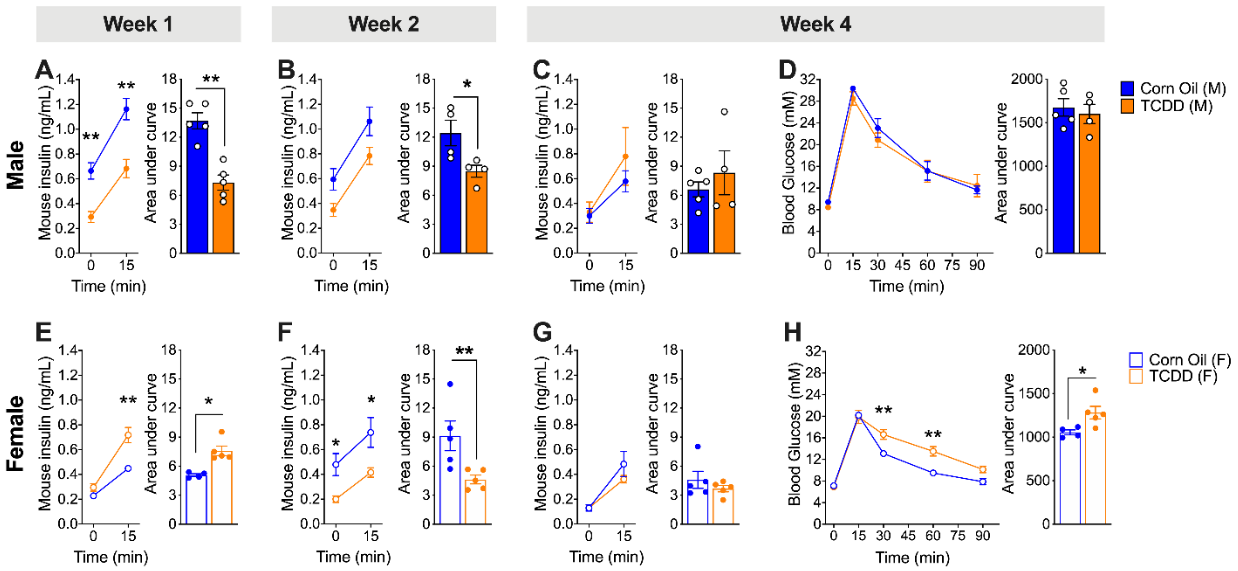

Figure S2: Transient TCDD exposure leads to supression of plasma insulin in both sexes, but sex differences in overall glucose tolerance *in vivo*. A new cohort of **(A-D)** male (M) and **(E-H)** female (F) mice were injected with either corn oil or 20 µg/kg TCDD on day 0 and glucose tolerance and glucose-stimulated insulin secretion was assessed *in vivo* on days 7, 14, and 28 (see Figure 3 for other cohort). **(A-C,E-G)** Plasma insulin levels before (time 0) and 15 minutes after a glucose injection on days 7 **(A,E),** 14 **(B,F),** and 28 **(C,G). (D,H)** Blood glucose levels were measured during a GTT on day 28. All data are presented as mean ± SEM. Individual data points on bar graphs represent biological replicates (different mice). *p<0.05, **p<0.01 versus control. The following statistical tests were used: **(A-H)** line graphs, two-way RM-ANOVA with Sidak test; (**A-D,H)** bar graphs, unpaired two-tailed t-test; **(E-G)** bar graphs, Mann-Whitney test.
